# Rheumatism and Gout

**Published:** 1901-03-30

**Authors:** 


### RHEUMATISM AND GOUT.

**Acute Rheumatism.**—Newsholme[1] argues that it is an epidemic disease, most frequent when a series of dry years occurs and rarer in wet ones when its supposed saprophytic organism is drowned out in the soil. Possibly the infection is conveyed to the human being by dust or vermin. The old idea that damp places, wet weather and clay soils increase acute rheumatism is untrue. On the other hand, though dry seasons with a low level of ground water show much rheumatism, it is not to be thought that artificial drainage with a permanent low level of the water ever increases it. As to the pathology Poynton[2] remarks on the absence of suppuration, though cell destruction and exudation and fibrosis show the local effects of some poison. The most noticeable point about the changes is the great local resistance and the tendency to recovery. As regards the lactic and uric acid theories the blood is not acid in rheumatic fever, and neither lactates nor an excess of uric acid have been found in it. The malarial and pus coccus theories are equally unfounded, but the writer and Alexander Paine have isolated in 12 successive cases a diplococcus which produced the symptoms when inoculated into rabbits, and which grows vigorously in a slightly acid mixture of milk and bouillon. It was found in pericardial fluid and exudation, urine, in the tonsils, in nodules and on the cardiac valves. It appears to be rapidly destroyed by the human tissues, and in unsuitable conditions developes a large monococcal type which is probably a resting form difficult to destroy. The diplococcus when growing actively forms "streptococcal" chains, and is probably the same as that found by Triboulet and Wasserman. E. F. Grün[3] claims to have also discovered it in 1892, and continues giving salicylates in acute rheumatism until it can be no longer seen in the blood to obviate the danger of relapses. In another article in the *Practitioner*, St. Clair Thompson[4] gives an account of the views which have been taken as to the relations between tonsillitis and acute rheumatism. Some writers have taught that the rheumatic poison enters through the tonsil, others that tonsillitis is merely one of the manifestations of the rheumatic diathesis. The present paper concludes that our knowledge is insufficient to decide the question; still some 30 to 80 per cent. of all cases of acute rheumatism are preceded by some form of angina, and the tonsil may possibly be the point of entry of the poison. Quinsy or abscess about the tonsil does not seem to be connected with rheumatism, and is not benefited by rheumatic remedies, unlike some other tonsillar affections. The cricothyroid joint is at times attacked by rheumatism.

G. A. Gibson[5] argues that heart affections are no more complications of the disease than the joint ones are, and that indeed the heart must be considered as one of the joints in its relation to the poison. In the treatment complete rest and light unstimulating food with salicylates, and then absorbents such as iodide of sodium in 10 or 15 grain doses should be given, and digitalis or strophanthus omitted. He also recommends flying blisters as employed by Caton. Luff[6] thinks that the charge against salicylate treatment with respect to heart lesions is due to its relieving other symptoms so rapidly that we are apt to relax our measures while the disease is in full force. Statistics from St. George's Hospital showed that under 2 per cent. of patients treated with alkalies and salicylates developed heart lesions, while 9·6 per cent. of those on salicylates alone did so. A milk diet with broth and vegetable soups, but no strong meat extracts, and copious diluent drinks should be given. He gives 20 grains of pure sodium salicylate and 30 of potassium (? sodium) bicarbonate every two hours until pain is relieved, and afterwards at longer intervals. If this is not well borne, or if the patient is a child, salicin is substituted for salicylates.

Obstinate pain in the joints may be treated by small blisters near them, which should be covered with a dressing and ointment firmly fastened down as soon as the blister is taken off. Salicylate of methyl on lint may be applied to a joint, and covered with gutta-percha tissue, which should be hermetically sealed by chloroform to prevent evaporation. Complete rest in bed should be maintained for six weeks, or, if cardiac symptoms have occurred, for eight weeks from their appearance, even though the salicylates have removed the pain in a few days. If cardiac failure occur, brandy, strychnine, and ammonia, and not digitalis should be given. For hyperpyrexia, cold baths must be relied on.

In children we find the usual symptoms replaced by "growing pains," heart lesions, nodules, or chorea, either together or separately, and cases of the disease probably amount to a quarter at least of all our hospital patients from two to ten years of age. G. F. Still[7] notices that frequently pain in one hip or at the back of the knee may be the only symptom for a time, and that cardiac dilatation or rapid action, wasting and nodules, pain in the stomach or in one axilla, headache, nervousness, night terrors and habit spasm are very frequent. Rheumatism seems specially apt to attack children with red hair, though statistics on the point are needed. Caton[8] claims that under his plan of treatment cardiac troubles can be reduced to 1·5 per cent. He forbids any excitement, does not even allow the head to be raised, keeps the patient in bed for six weeks or more, and applies a succession of small blisters between the clavicle and the nipple, besides giving iodides and even mercury during convalescence. W. H. Porter[9] regards gout and rheumatism as due to various degrees of deficient oxidation in the tissues, produced by the absorption from the intestinal canal of certain toxic products, which are themselves the result of microbic action.

D. F. Battistini,[10] in speaking of the treatment of painful joints, refers to methyl salicylate which may be made into an ointment to obviate the odour. Salicylic acid is apt to redden the skin and cause desquamation, but is rapidly absorbed, especially if made up with an animal fat such as lanolin. Salol, menthol, ether, and lanolin as an ointment sometimes gives remarkable results in gonorrhœal rheumatism, and in chronic joint troubles icthyol may be added to lanolin, turpentine, and oil of wintergreen. Von Rottenbiller speaks highly of this oil of wintergreen, given internally in gelatin capsules to the amount of 90 drops a day, and reports 122 cases in which he used it. There were acute and chronic rheumatisms, gonorrhœal rheumatism, and arthritis deformans. Even if it fails to cure it affords much relief from pain.

[1] Practitioner, Jan. [2] Loc. cit. [3] Lancet, Oct. 13th. [4] Practitioner, Jan. [5] Loc. cit. [6] Loc. cit., p. 64. [7] Loc. cit. [8] Brit. Med. Jour., Oct. 20th. [9] Med. Rec., Sept. 22nd. [10] Amer. Jour. Med Sciences, Dec.

